# Phase I/IIa study of concomitant radiotherapy with olaparib and temozolomide in unresectable or partially resectable glioblastoma: OLA-TMZ-RTE-01 trial protocol

**DOI:** 10.1186/s12885-019-5413-y

**Published:** 2019-03-04

**Authors:** Paul Lesueur, Justine Lequesne, Jean-Michel Grellard, Audrey Dugué, Elodie Coquan, Pierre-Emmanuel Brachet, Julien Geffrelot, William Kao, Evelyne Emery, David Hassanein Berro, Laurent Castera, Nicolas Goardon, Joëlle Lacroix, Marie Lange, Aurélie Capel, Alexandra Leconte, Benoit Andre, Angélique Léger, Anaïs Lelaidier, Bénédicte Clarisse, Dinu Stefan

**Affiliations:** 1 Radiation Oncology Department, Centre François Baclesse, 3 avenue du Général Harris, F-14076 Caen, France; 2 Normandy university, University of Caen Basse Normandie, 14000 Caen, France; 3 Clinical Research Department Centre François Baclesse, Caen, France; 4 Medical Oncology Department, Centre François Baclesse, 14076 Caen, France; 5 Neurosurgery Department, CHU de Caen, Caen, France; 6 Genetic and oncology biology Department, Centre François Baclesse, Caen, France; 7 Radiology Department, Centre François Baclesse, Caen, France; 8 UNICAEN, INSERM, U1086 Anticipe, Normandie University, 14076 Caen, France; 9 IT department, Centre François Baclesse, F-14000 Caen, France; 10 Pharmacy, Centre François Baclesse, F-14000 Caen, France; 11 Northwest Data Center (CTD-CNO), Centre François Baclesse, F-14000 Caen, France

**Keywords:** Glioblastoma, Poly (ADP-ribose) polymerase (PARP), Radiotherapy, Radiosensitizer, olaparib

## Abstract

**Background:**

Despite multimodality treatments including neurosurgery, radiotherapy and chemotherapy, glioblastoma (GBM) prognosis remains poor. GBM is classically considered as a radioresistant tumor, because of its high local recurrence rate, inside the irradiation field. The development of new radiosensitizer is crucial to improve the patient outcomes. Pre-clinical data showed that Poly (ADP-ribose) polymerase inhibitors (PARPi) could be considered as a promising class of radiosensitizer.

The aim of this study is to evaluate Olaparib, a PARPi, as radiosensitizing agent, combined with the Stupp protocol, namely temozolomide (TMZ) and intensity modulated radiotherapy (IMRT) in first line treatment of partially or non-resected GBM.

**Methods:**

The OLA-TMZ-RTE-01 study is a multicenter non-randomized phase I/IIa trial including unresectable or partially resectable GBM patients, from 18 to 70 years old. A two-step dose-escalation phase I design will first determine the recommended phase 2 dose (RP2D) of olaparib, delivered concomitantly with TMZ plus conventional irradiation for 6 weeks and as single agent for 4 weeks (radiotherapy period), and second, the RP2D of olaparib combined with adjuvant TMZ (maintenance period). Phase IIa will assess the 18-month overall survival (OS) of this combination. In both phase I and IIa separately considered, the progression-free survival, the objective response rate, the neurocognitive functions of patients, emotional disorders among caregivers, the survival without toxicity, degradation nor progression, the complications onset and the morphologic and functional MRI (magnetic resonance imaging) parameters will be also assessed as secondary objectives. Ancillary objectives will explore alteration of the DNA repair pathways on biopsy tumor, proton magnetic resonance spectroscopy parameters to differentiate tumor relapse and radionecrosis, and an expanded cognition evaluation. Up to 79 patients will be enrolled: 30 patients in the phase I and 49 patients in the phase IIa.

**Discussion:**

Combining PARP inhibitors, such as olaparib, with radiotherapy and chemotherapy in GBM may improve survival outcomes, while sparing healthy tissue and preserving neurocognitive function, given the replication-dependent efficacy of olaparib, and the increased PARP expression in GBM as compared to non-neoplastic brain tissue. Ancillary studies will help to identify genetic biomarkers predictive of PARPi efficacy as radiosensitizer.

**Trial registration:**

NCT03212742, registered June, 7, 2017. Protocol version: Version 2.2 dated from 2017/08/18.

## Background

Glioblastoma multiforme (GBM) is one of the most common primary brain malignant tumor in adults. The World Health Organization (WHO) classified gliomas into four grades of ascending malignancy [1]. According to this classification, GBMs are included in grade IV gliomas. They are very aggressive tumors, associated with a median overall survival (OS) around 3 to 6 months without any treatment.

The mainstay of treatment in these patients diagnosed with a GBM includes image-guided maximal surgical resection, followed first, by external beam radiotherapy delivered concomitantly with temozolomide (TMZ) and then subsequently by additional adjuvant TMZ cycles, named the Stupp protocol [2]. Despite significant improvements in neuro-imaging, chemotherapy, surgical and radiotherapy techniques, the prognosis for these patients is still poor, with a median OS of 14.6 months and an OS rate of 27% after 2 years, dropping then below 10% after 5 years [2]. Recurrence occurs in more than 90% of GBM patients, mostly in the irradiation field [3]. As a consequence, GBM is considered as a chemo- and radioresistant tumor. This radioresistance could be explained because of the presence of glioblastoma stem cells (GSCs). GSCs feature high DNA repair capability. GSCs are a specific subpopulation of GBM cells with properties of tumor stem cells, such as differentiation, unlimited proficiency, and self-renewal, as well as high DNA repair capacity, which explains a major part GBM radioresistance. In GSCs, proteins involved in DNA repair mechanism such as checkpoint kinase 1 (CHK1), ATM serine/threonine kinase (ATM), poly (ADP-ribose) polymerase 1 (PARP1), or ATR serine/threonine kinase (ATR), seem to be upregulated. Furthermore, GSCs exhibit resistance to drugs, including TMZ. One of the underlying hypothesis stated for explaining the poor GBM patient survival is that the GSCs population responds differently to TMZ treatment or radiation therapy than the non-stem cell population [4, 5]. Thus, GSCs and their high DNA repair capability could be considered as drugable targets.

In case of recurrence, there is no standard care strategy, and the clinician is often helpless.

As GBM surgery is a major prognostic factor, the maximum safe reduction of tumor burden should be attempted, even in situations in which a gross total resection cannot be achieved. Retrospective studies indeed showed that the gross total resection could improve OS [6, 7]. Unfortunately, the microscopic infiltration of the GBM or the tumor localization often render the total resection difficult, making the prognosis poorer for the patients with unresectable GBM [8].

Besides the Stupp protocol that remains the mainstay of treatment for newly diagnosed GBM patients, there is an emergency to find new therapeutic strategies to improve our patients outcomes.

PARP1 has an important cellular function, detecting the presence of damaged DNA and then activating signalling pathways that promote appropriate cellular responses. PARP1 is involved in base excision repair (BER), allowing the recruitment and activation of BER factors, and consequently facilitating the repair of DNA single-strand breaks (SSBs). PARP1, 2, and 3 are equally involved in other cellular mechanisms, such as chromatin remodelling and DNA double-strand break repair [9].

PARP inhibition has been therefore considered as a novel approach to target tumors with deficiencies in DNA repair mechanisms. It may enhance the DNA damaging effect of chemotherapy and ionizing radiation.

According to in vitro studies, inhibition of PARP would enhance the killing effect of TMZ, by inhibiting DNA base excision repair mechanism. PARP inhibitors (PARPi) first entered into clinical trials in 2003 in combination with the monomethylating agent TMZ for patients with advanced solid tumors [10, 11]. Their development has been accelerated since the concept of synthetic lethality appeared in homologous recombination-deficient cells that were exposed to PARPi [12, 13].

PARPi have been also considered as potential radiosensitizers considering its ability to increase the number of unrepaired DNA double strand breaks (DSBs). The radiosensitizing effect of PARPis is S-phase dependent [14]. This replication-dependent mechanism allows adjacent healthy tissues to be spared by the radiosensitizing effect while highly proliferative tumors would be sensitized. To date, more than a dozen of preclinical studies and one phase I trial have investigated PARPi as radiosensitizers for glioma cells. The Enhancement ratios issued from pre-clinical studies ranged between 1.08 and 1.93 [11], and the only full published phase I trial assessed the safety of a PARPi (veliparib) combined with irradiation [15]. Preclinical data are then promising and PARPi could be considered as new radiosensitizer agent [16].

Thus, recently, results from two phase I trial with olaparib in GBM patients were very promising. Olaparib is a PARPi developed as an oral therapy, alone or in combination, with chemotherapy. On the first hand, the OPARATIC trial associating olaparib and TMZ in patients with relapsed GBM showed that olaparib penetrates core and margins of recurrent GBM [17]. It also highlighted that olaparib combined with TMZ requires intermittent dosing but is safe and well tolerated. One the other hand, olaparib combined with radiotherapy, assessed among newly diagnosed GBM, showed this combination is extremely well tolerated (PARADIGM trial) [18].

To our knowledge, excepted PARADIGM Trial preliminary results, none ongoing protocol has assessed the association of a PARPi, TMZ and radiotherapy in first line treatment of unresectable or partially resected GBM.

Assuming that PARP inhibition should overcome treatment resistance of GBM, through a specific radiosensitization of proliferating cells, and an enhancement of DNA damages induced by chemotherapy, we propose a phase I-IIa study to evaluate olaparib in combination with TMZ and radiotherapy (Stupp protocol) for first line treatment of patients with partially resected or unresected GBM.

## Methods/design

### Primary outcome

The primary purpose of the phase I part of OLA-TMZ-RTE 01 Study is to determine the Recommended Phase II Dose (RP2D) of olaparib combined with the standard schedule of radiotherapy and temozolomide as first line treatment of patients with unresectable GBM. For the phase II part, the main objective is to assess the 18-months OS of patients with unresectable GBM treated with olaparib combined with Stupp protocol.

### Secondary outcomes

The secondary objectives are to evaluate:

- The progression-free survival,
- The objective response rate,
- The neuro-cognitive functions and quality of life of patients and emotional disorders among caregivers
- The survival without toxicity, without degradation (quality of life and / or cognitive disorders) nor tumor progression (Q-TWIST for Quality-Adjusted Time Without Symptoms or Toxicity) and the prevalence of treatment induced complications,
- The evolution of corticosteroids use
- The morphologic and functional MRI parameters
- If alterations of DNA repair ways in GBM cells could predict response to the therapeutic combination.

### Eligibility criteria

Patients have to fulfill the following main inclusion criteria:

- Histologically-confirmed diagnosis of GBM (IDH-wildtype, IDH-mutant or NOS, except gliosarcoma), non resectable or partially resectable with a residual tumor on pre-radiotherapy MRI. The presence of a residual disease will be assessed by the radiologist on the pre-radiotherapy imaging as compared with initial imaging,
- IMRT must start within 6 weeks after histological diagnosis;
- age between 18 and 70 years;
- neurologically asymptomatic or pauci-symptomatic patients. Patients with moderated neurological symptoms without systemic corticosteroids treatment or with a stable dose of corticosteroids during the study as long as these were started at least 4 weeks prior to treatment can be included;
- adequate bone marrow and organ function measured within 15 days prior to administration of study;
- ECOG performance status 0–2
- patients must have a life expectancy ≥16 weeks.
- Written informed consent before and study-related procedure

Main criteria to exclude subjects from the trial are:

- any prior radiotherapy to brain;
- any prior chemotherapy or immunotherapy;
- candidate for a concomitant therapy with Tumor-Treating Fields during the maintenance treatment;
- any previous treatment with a PARP inhibitor, including olaparib;
- patients who had no initial pre-surgery contrast enhanced MRI scan including the standard sequences (T1 non enhanced, T1 contrast enhanced, T2 FLAIR);
- patients receiving any systemic chemotherapy, radiotherapy (except for palliative reasons), within 2 weeks from the last dose prior to study treatment;
- concomitant use of known strong CYP3A inhibitors;
- concomitant use of known strong or moderate CYP3A inducers.

Patients discontinuating.

### Study treatments

The therapeutic regimen will be divided into 2 different periods:

- a radiotherapy period
- a maintenance period.

For the radiotherapy period, the patient will start IMRT (60Gy/30fr/6 weeks), TMZ chemotherapy (75 mg/m^2^/day), and olaparib on the same day, on a Monday (day 1), within 6 weeks after surgery. The daily dose of TMZ (75 mg/m^2^) will be continued until the end of radiotherapy (6 weeks) and olaparib will be continued with the same dose until 4 weeks after the end of IMRT, as a single agent. Then, TMZ will be re-introduced 4 weeks after the end of IMRT at the dose of 150 mg/m^2^/day on days 1 to 5 every 28 days, for a total of 6 cycles, according to the standard Stupp protocol.

For the maintenance period, olaparib will be given at the maintenance dose level up to confirmed disease progression or unacceptable toxicities. It concerns only the second part from the phase I and the entire phase IIa.

### Study sites

The list of study sites is available on https://clinicaltrials.gov/ct2/show/NCT03212742

### Trial schedule and statistical design overview

The OLA-TMZ-RTE 01 study is a multicenter non-randomized phase I/IIa trial.

Seven dose levels of olaparib will be assessed in two periods by two sequential dose-escalations to define the RP2D of olaparib in combination with the Stupp protocol:

- The maximum tolerated dose MTD1 corresponding to the dose of olaparib during the radiotherapy period: from day 1 of radiotherapy up to 4 weeks after the end of irradiation; a maximum of 15 assessable patients will be enrolled for this first dose-escalation;
- The MTD2 corresponding to the dose of olaparib in the maintenance period: from 4 weeks after radiotherapy, at the time of re-introduction of temozolomide, until progression; a maximum of 15 assessable patients will be enrolled for the second dose-escalation.

Then, the efficacy of this treatment combination, in terms of the 18-month OS rate, will be assessed in a Phase IIa step using a two-stage Case & Morgan design [19], requiring 44 assessable patients (including 30 patients in the first stage). To anticipate some non assessable patients (10% of enrolled patients), we have planned to enroll 33 patients in the first stage and 16 in the second, for a total of 49 patients.

To conclude, 30 patients will thus be enrolled in the phase I part and 49 in the phase IIa part, leading to a total of 79 patients for both phases.

### Phase I part

Based on the phase II study Stupp et al. [20] design, the period of DLT is split in order to propose the best olaparib dose level during the radiotherapy period, with the possibility to reduce this dose level during the maintenance period.

**Fig. 1:**
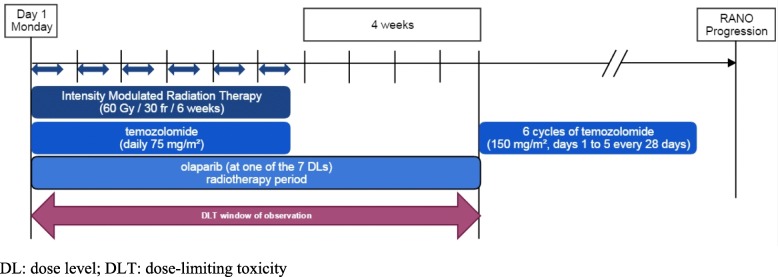
Dose escalation during the radiotherapy period (OLA-TMZ-RTE 01 trial)

**Fig. 2:**
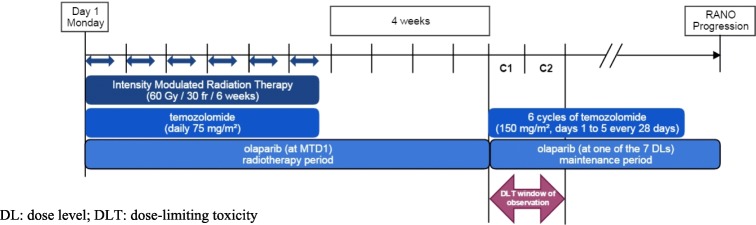
Dose escalation during the maintenance period (OLA-TMZ-RTE 01 trial)

We thus propose to conduct two sequential dose-escalations of olaparib:

- Patients from the first dose-escalation will receive olaparib during 10 weeks, including the radiotherapy period (6 weeks) and the 4 following weeks until the start of the adjuvant TMZ in order to determine the MTD1 by a TITE-CRM [21, 22] (TIme-To-Event Continual Reassessment Method) modeling DLT observed during the radiotherapy period (Fig. 1).
- Patients from the second dose-escalation will receive olaparib at the MTD1 during the radiotherapy period, and then a dose-escalation of olaparib during maintenance period will be done, also by a TITE-CRM modeling. DLTs observed during the first two cycles of the maintenance period will be used to determine the MTD2 (Fig. 2).

For both dose-escalations, a minimum of 4 consecutive patients will have to be treated at the MTD before being ended.

### Phase II part

According to a Case & Morgan design the study may stop for futility at interim analysis or conclude to inefficacy at final analysis considering the following assumptions:

- H0: the overall survival rate at 18 months (18-mo OS) ≤ 23.9% *(corresponding to the upper limit of the 2-y OS rate of 14.8% with IMRT alone* [2] *under the assumption of an exponential distribution)*
- H1: 18-mo OS ≥43% *(corresponding to the upper limit of the 2-y OS rate of 32.5% obtained with the Stupp protocol* [2] *under the assumption of an exponential distribution.)*

Any assessable patient receiving IMRT, TMZ and Olaparib at the RP2D (determined from the phase I part) on the first day will be considered for evaluating efficacy. OS is defined as the delay from treatment initiation to death from any cause, with surviving patients censored at last follow-up. The null hypothesis H0 will be tested using the asymptotically standard normal test statistic (Z) computed from the log of the Nelson-Aalen estimator for the cumulative hazard of death. At the interim analysis, that will be performed once included the 33rd patient at the RP2D, the test statistic Z1 will be compared to the lower bound C1L: if Z1 < = C1L (C1L = -0.179), the study may be stopped for futility, concluding that the association of Olaparib, TMZ and IMRT is not efficacious enough for treating unresectable GBM in first line. Otherwise the study will continue to the second stage. At the final analysis, which is foreseen 18 months after the later inclusion, the test statistic Z2 will be compared to the upper bound C2U (C2U = 1.233): if Z2 > =C2U, we will reject H0 and conclude to efficacy. The sample size is computed according to an overall alpha risk of 10% to achieve a power of 90%.

### Planned dose levels of olaparib

Seven dose levels are defined to reach the target dose of 400 mg per day (200 mg twice daily) of olaparib continuously (Table 1).

**Table 1:** Dose levels for the phase I part of the OLA-TMZ-RTE trial

DL (Dose Level)	Olaparib (orally)	STUPP protocol	
		Temozolomide	IMRT
	Each treatment will start on a Monday		
DL0	50 mg Q12H, Monday to Tuesday	• 75 mg/m^2^/day until end of IMRT • 4 weeks after the end of IMRT: 6 cycles of temozolomide at the dose of 150 mg/m^2^/day on days 1 to 5 every 28 days	• IMRT will begin at day 1 • Prescription doses will be 60Gy/30fr/6 weeks • The PTV receives 95 to 107% of the prescribed dose
DL1 (starting dose level)	50 mg Q12H, Monday to Wednesday		
DL2	100 mg Q12H, Monday to Wednesday		
DL3	100 mg Q12H, Monday to Friday		
DL4	200 mg Q12H, Monday to Wednesday		
DL5	200 mg Q12H, Monday to Friday		
DL6	200 mg Q12H, continuously		

### Concept of TITE-CRMs

TITE-CRMs allow to take into account delayed toxicities, by weighting the period of observation, without the need to wait until the end of the DLT observation period before enrolment of a new patient [21, 22]. However, the knowledge about toxicities potentially induced by the combination of PARP inhibitors, TMZ and radiotherapy in first line treatment of unresectable GMB, is quite limited. Thus the TITE-CRM method used in this study has been adapted as follows:

- For each dose level not yet explored, the first patient will be observed during 10 weeks before enrolling a new patient. In the absence of previous DLT at this dose level, the following patients at this dose level will be observed a minimum of 3 weeks before each enrollment.
- One can escalate to an upper level if and only if the previous level has already been explored. Otherwise, the dose level assigned to the next patient is the first dose level upper than the maximal dose level explored in the dose-escalation.

The MTD of each period of treatment will be defined as the highest dose level of olaparib with a proportion of patients experiencing DLT within this period less than θ (θ = 25 and 30% for the radiotherapy and maintenance period, respectively). The vector of initial estimates of toxicity probabilities associated to the dose levels is p = (0.05,0.10,0.20,0.25,0.30,0.35,0.40), expecting relatively high MTDs at the end of the dose-escalation. The R2PD will be defined as the MTD1 in the radiotherapy period and the MTD2 in the maintenance period.

### Study procedures

The overview of study assessments and procedures are detailed in Table 2.

**Table 2:** Overview of study assessments of the OLA-TMZ-RTE-01 trial

	Screening/Baseline	Radiotherapy period											Maintenance period (TMZ + Olaparib)													Post-treatment Visit^e^	Follow up every 2 months
		IMRT						Treatment break					Cycle 1				Cycle 2				Cycle 3 to 6				Olaparib maintenance		
Week (W)		W 0	W 1	W 2	W 3	W 4	W 5	W 6	W 7	W 8	W 9	W 10	W 11	W 12	W 13	W 14	W 15	W 16	W 17	W 18	W 19	W 23	W 27	W 31	W 35		
Day	(−28 to −1)	1	8	15	22	29	36	43	50	57	64	71	78	85	92	99	106	113	120	127	134	162	190	218	246		
Informed consent	✓																										
Medical history and demographic	✓																										
Initial histology	✓																										
Prior/ concomitant medication (corticosteroid)	✓			✓																							
Adverse event evaluation	✓			✓																							
Contrast enhanced MRI	✓								✓				✓								✓^a^		✓^a^		✓^a^	✓^a^	✓^a^
Ancillary spectro-MRI^b^	✓								✓				✓								✓^a^		✓^a^		✓^a^	✓^a^	✓^a^
Dosimetric Brain CT-scan	✓																										
Neurological assessment	✓	✓	✓	✓	✓	✓	✓	✓	✓				✓	✓	✓	✓	✓	✓	✓	✓	✓	✓	✓	✓	✓	✓	✓ ^d^
Physical examination, weight, vital signs	✓	✓	✓	✓	✓	✓	✓	✓	✓				✓	✓	✓	✓	✓	✓	✓	✓	✓	✓	✓	✓	✓	✓	✓
Performance status OMS	✓	✓	✓	✓	✓	✓	✓	✓	✓				✓	✓	✓	✓	✓	✓	✓	✓	✓	✓	✓	✓	✓	✓	✓
ECG	✓																										
Serum pregnancy test	✓																										
Hematology	✓	✓	✓	✓	✓	✓	✓	✓	✓	✓	✓	✓	✓	✓	✓	✓	✓	✓	✓	✓	✓	✓	✓	✓	✓	✓	
Fasting blood chemistry	✓	✓		✓		✓		✓	✓		✓		✓		✓		✓		✓		✓^c^	✓^c^	✓^c^	✓^c^	✓^c^	✓	
Coagulation (INR, aPTT)	✓																										
Urinalysis	✓																										
Ancillary biomarker exploration^b^ -biopsy of primitive tumor -blood sample	✓																										
Cognition and Quality of Life (QoL)																											
-MoCA EORTC QLQ-C30 + BN20	✓												✓										✓^d^				
-Ancillary assessments^b^	✓												✓										✓^d^				
Radiation (IMRT)			2Gy*5 days/week for 6 weeks																								
Temozolomide			75 mg/m^2^ daily from first to last day of IMRT										5 days				5 days				5 days	5 days	5 days	5 days			
Olaparib			Cf dose level						Cf dose level																		
Drug accountability			✓	✓	✓	✓	✓	✓					✓	✓	✓	✓	✓	✓	✓	✓	✓	✓	✓	✓			
Morbidity, survival		✓																									

^a^ Every 8 weeks

^b^ For voluntary patients and their caregivers with signed ancillary consents

^c^ Every 4 weeks

^d^ At Day 1 of cycle 5

^e^ For patients without disease progression

### Brain tumor evaluation

It will be performed on a 1.5 Tesla MRI machine at baseline, at the end of radiotherapy, 4 weeks after the end of radiotherapy and thereafter every 8 weeks in the absence of tumoral progression. It will include the realization of the axial T2* and single voxel spectroscopy sequences. Disease assessment evaluation will be determined locally according to RANO (Response Assessment in Neuro-Oncology) criteria [23].

### Neurological and quality of life assessment

A cognitive screening Montreal Cognitive Assessment (MoCA) and a Quality of life assessment (EORTC QLQ-C30 self-administered questionnaire and its specific brain cancer module (BN-20)) will be performed at baseline, 4 weeks and 5 months post radiotherapy if no disease progression has occurred. Additional cognition and quality of life (QoL) evaluation will be realized for voluntary patients.

### Ancillary studies

The protocol includes ancillary studies on tumor biopsies, spectro-MRI, neurocognitive and QoL assessment before and after IMRT. Each of these ancillary studies will be realized for voluntary patients having given specific consent.

### Biological ancillary study

A collection of blood samples and tumor biopsy will be constituted for ancillary biological study. In GBM, an increase of the radio-sensitization in association with olaparib could be observed in tumor with DNA repair mechanisms alteration. We therefore propose a comprehensive approach, integrating DNA sequencing (exome sequencing and CNV analysis with new generation sequencing epigenome analysis (methylation analysis) together with immunohistochemistry performed on collected biopsies to identify the tumors presenting an alteration explaining a sensitivity or resistance. A specific focus will be made on the DNA repair pathways and to the methylation status of the MGMT promoter to identify patient sensible or resistant to TMZ. This ancillary study aims to analyze the molecular tumor profile, assessing the prognostic impact of the different alterations comparing with therapeutic responses based on genetic profiles. The molecular analyses will be performed on the more recent tumor sample representative of the primitive GBM tumor. Immunohistochemistry (IHC) will be performed on biopsy to assess foci of proteins involved in the homologous repair/fanconi anaemia pathways: RAD51, BRCA1, MRE11and FANCD2; hallmarks of the repair pathways: γH2AX, Ub and PAR abundance to evaluate PARP suractivation.

### MRI ancillary study

In addition to the standard MRI imaging protocol, each MRI imaging evaluation will include a multivoxel spectroscopy imaging (MSI). A slice choice for exploration volume positioning will be determined by the referent radiologist from each investigator center. Exploration volume should include the primitive tumor, residual tumor or treated zone, and surrounding environment. Only one slab will be explored by spectroscopy imaging, and should be reproducible positioned for each patient. Evaluations may be helpful to explore the biochemistry of the tumor. Indeed, it appears important to be able to differentiate a tumor relapse from a therapeutic effect (radionecrosis) in the setting of this therapeutic association.

### Neurocognitive ancillary study

For each enrolled patients, cognition and health-related QoL will be assessed at baseline, 4 weeks after the end of IMRT and at day 1 of cycle 5 (unless the patient progressed).

In addition, as part of a more extended scientific program in our institution assessing various treatment approaches in brain tumors, a comprehensive assessment of cognition will be performed. This will allow to better assess the impact of adding olaparib to the Stupp protocol on cognition and QoL. The emotional disorders of patients’ caregivers will be as well explored at the same times in order to document the impact of altered cognitive performances and quality of life for GBM patients on informal caregivers.

This ancillary study will be performed in some participating centers by a dedicated neuropsychologist.

### Data management

A Web Based Data Capture (WBDC) system will be used for data collection and query handling. The investigator will ensure that data are recorded on the eCRFs as specified in the study protocol and in accordance with the instructions provided.

The investigator ensures the accuracy, completeness, and timeliness of the data recorded and of the provision of answers to data queries according to the Clinical Study Agreement. The investigator will sign the completed eCRFs. A copy of the completed eCRFs will be archived at the study site.

### Data monitoring comitee

An Independent Data Monitoring Committee (IDMC) will be set-up to ensure the protection of patients, the ethical conduct of the study, to evaluate the benefit/risk ratio of the study, and to insure an independent review of the scientific outcomes during and at completion of the study. The IDMC exercises a consultative role for the promoter who takes the final decision for implementing the recommendations proposed by the IDMC. The committee will include a radiotherapist, an oncologist, a statistician and a pharmacologist.

### Withdrawal from study

Reasons a patient may discontinue treatment include, but are not limited to:

- Treatment failure/confirmed disease progression (at 2nd MRI)
- Major protocol violation
- Intolerable toxicity
- Concomitant disease or other reason requiring the discontinuation of treatment
- Patient request (withdrawal of consent for further treatment)
- Investigator’s request (with detailed documentation of reasoning)
- Non-compliance of patient
- Trial termination by the sponsor
- Pregnancy
- Death

## Discussion

Given the poor prognosis of high risk GBM, the interest of IMRT techniques for sparing healthy tissue, the replication dependent efficacy of PARPi, and the increased PARP expression in GBM as compared to non-neoplastic brain tissue, a combination of PARP inhibitors with radiotherapy and chemotherapy in newly diagnosed unresectable or partially resected GBM represents a promising way to improve care for these patients.

We propose a study aiming at assessing the interest of olaparib combined with the irradiation and TMZ, which remains the standard of treatment for newly diagnosed unresectable GBM. Such a trial is an opportunity for patients to benefit from an innovating treatment. In addition, to improve the knowledge on the deleterious clinical symptoms of GBM, we plan to perform some additional investigations in brain imaging as well as in cognitive functioning and quality of life. Furthermore, we propose an original molecular comprehensive approach, integrating targeted DNA sequencing (mutation and CNV analysis), epigenome analysis (methylation analysis) together with immunohistochemistry performed on collected biopsies in order to identify the tumors presenting an alteration of the DNA repair pathways.

## Abbreviations

ATM: ATM serine/threonine kinase
ATR: ATR serine/threonine kinase
BER: Base excision repair
CHK1: Checkpoint kinase 1
CNV: Copy number variant
DLT: Dose limiting toxicities
DNA: Deoxyribose nucleic acid
GBM: Glioblastoma multiforme
GSCs: Glioblastoma stem cells
HGG: High-grade gliomas
HRD: Homologous Recombination Deoxyribonucleic acid Repair
IMRT: Intensity modulated radiotherapy
MoCA: Montreal Cognitive Assessment
MRI: Magnetic resonance imaging
MTD: Maximum-tolerated dose
OS: Overall survival
PARP: Poly (ADP-ribose) Polymerase
PARPi: Poly (ADP-ribose) Polymerase inhibitor
QoL: Quality of life
Q-TWIST: Quality-adjusted time without symptoms or toxicity
RANO: Response Assessment in Neuro-Oncology Criteria
RP2D: Recommended phase 2 dose
SSBs: Single-strand breaks
TITE-CRM: Time-to-event continual reassessment method
TMZ: Temozolomide
WHO: World Health Organization
